# Does the Use of Intraoperative Breast Sizers Increase Complication Rates in Primary Breast Augmentation? A Retrospective Analysis of 416 Consecutive Cases in a Single Institution

**DOI:** 10.1155/2016/6584810

**Published:** 2016-03-22

**Authors:** Lee Seng Khoo, Henrique N. Radwanski, Vasco Senna-Fernandes, Nsingi Nsosolo Antônio, Leonardo Luiz Fernandes Fellet, Ivo Pitanguy

**Affiliations:** Instituto Ivo Pitanguy, Rua Dona Mariana 65, 22280-020 Botafogo, RJ, Brazil

## Abstract

*Background.* Is the use of intraoperative breast sizers beneficial for plastic surgeons or do they result in higher complication rates?* Methods.* This is a retrospective study of 416 consecutive cases of primary breast augmentation with silicone implants at the Plastic Surgery Service of Professor Ivo Pitanguy at the 38th Infirmary Santa Casa Misericórdia Hospital, Rio De Janeiro, from January 2011 to March 2014. 212 cases (51%) were carried out with use of intraoperative breast sizers with 204 cases (49%) without the use of implant sizers. This study compares the outcome of cases that employed the use of intraoperative implant sizers versus those that did not in terms of infection, hematoma/seroma formation, and capsular contracture.* Results.* Of 416 primary breast augmentation cases, there were 5 cases of infection (1.2%), 4 cases of seroma (1%), 3 cases of hematoma (0.7%), and 7 cases of capsular contracture (Baker's Grade III/IV)(1.7%). Total complication rate limited to infection, seroma, hematoma, and capsular contracture was 1.15% (95% CI 0.96–1.93%). There was a significant difference in the scores for breast sizers (M = 4.3, SD = 1.4) and no breast sizers (M = 2.3, SD = 0.87) conditions, *t*(8) = 2.79, *p* = 0.018. The use of implant sizers was correlated with a higher complication rate.* Conclusion.* Good results could be obtained without the use of breast sizers in primary breast augmentation with use of a biodimensional tissue based planning system while eliminating risks of infection and reducing intraoperative time. Notwithstanding, in a residency program breast sizers can be an excellent training tool to shorten the learning curve in the novice surgeon.

## 1. Introduction

Many plastic surgeons utilize breast implant sizers in breast augmentation surgery to estimate the ideal implant volume after pocket dissection.

Intraoperative breast implant sizers are sterilizable and reusable and also provide a visual gauge of volume required to adequately fill the breast pocket.

However, the routine usage of these implant sizers may cause tissue trauma, augment contamination risks, and increase intraoperative time and may also be expensive. In the United Kingdom, implant sizers are mandated to be single use apparatus that must be discarded after one surgery [1].

**Figure 1:**
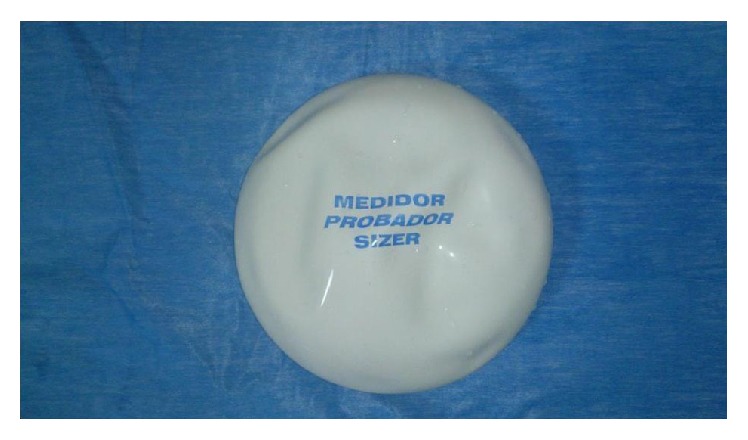
An example of an intraoperative sizer. Note that the sizer does not have the consistency of an implant.

Many sizers including tissue expanders do not accurately simulate the shape surface characteristics of the implant and therefore do not accurately reflect the visual appearance that a similar volume implant may produce [2, 3] (Figure 1).

**Figure 2:**
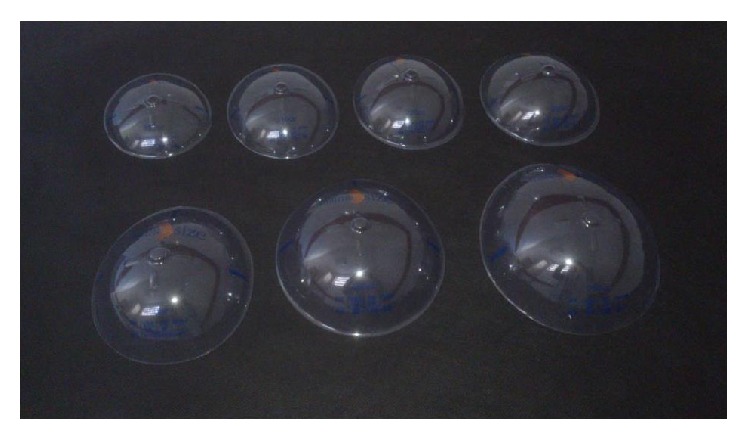
External breast sizers in various sizes.

The alternative to intraoperative breast sizers such as trial sizing with external breast sizers (Figure 2) or stockings filled with rice bags in the consultation room [4, 5] is also notoriously inaccurate in determining the ideal implant or visual representation.

Surgeons such as Tebbetts and Heden have developed mathematical preoperative breast tissue based planning to eliminate the use of implant sizers [2, 3].

Anecdotally, surgeons have been performing preoperative planning subjectively at best and often not even included prior to the surgical procedure. The premise of a tissue based preoperative algorithm such as the Akademikliniken method [3] and High Five system of Tebbetts [6] is to evaluate the patients' tissues and objectively match an implant specifically to what the tissues can accommodate.

Case series of 2500 primary breast augmentations performed with tissue based preoperative planning in the United States demonstrated a reoperative rate of 3% within 6 to 7 years' follow-up, compared to the reoperation rate of 15% to 20% in 3 years in all the PMA (Premarket Approval) implant case series [7–10].

Nonetheless, the use of breast implant sizers continues to be popular among surgeons [7–10]. Gore reported that less than 2% of patients in consecutive 200 case series developed complications such as hematoma, seroma formation, or infection [11]. Capsular contracture was noted in 7% of patients, but there were no visible or painful capsules [11]. A 5-year study detailing reoperative augmentation mammaplasty by Pitanguy et al. revealed complication rates of primary breast augmentation comparable to other studies [12]. Pitanguy described his preferred route for placing breast implants via a transareolopapilar incision [13] and utilized external breast sizers to determine the ideal volume necessary to obtain a satisfactory result [14].

The question therefore to be asked is whether the routine use of breast implant sizers is necessary in primary breast augmentation. Is there a higher rate of complications such as hematoma, seroma, infection, and capsular contracture in patients that undergo breast augmentation with sizers compared to those who did not?

## 2. Materials and Methods

We present a retrospective study of 416 consecutive cases of primary breast augmentation with silicone implants at the Plastic Surgery Service of Professor Ivo Pitanguy at the 38th Infirmary Santa Casa Misericórdia Hospital, Rio de Janeiro, from January 2011 to March 2014. This study compares the outcome of cases that employed the use of intraoperative implant sizers versus those that did not in terms of infection, hematoma/seroma formation, and capsular contracture.

Inclusion criteria included all consecutive patients who underwent primary breast augmentation with or without the use of intraoperative implant sizers within the period of January 2011 to March 2014. Patients who also had simultaneous mastopexy (augmentation mastopexy), secondary augmentation mammaplasty, change of implants and reconstructive breast surgery with implants, and combined surgeries with other procedures were excluded from this study.

Accordingly we sought to measure complications that can be attributed to the use of intraoperative sizers per se such as infection, hematoma/seroma formation, and capsular contracture. We excluded technique/surgeon related complications such as inadequate size, breast asymmetry, implant malposition, implant palpability, and wrinkles, ripples, or folds seen in the breast tissue and the skin overlying the implant.

A list of all patients who underwent breast augmentation with implants from January 2011 to March 2014 was located from the archive database at the 38th Infirmary Santa Casa Misericordia Hospital. After applying the exclusion criteria we were left with a total of 416 patients that underwent primary breast augmentation from January 2011 to March 2014, 212 with intraoperative sizers and 204 without intraoperative sizers. Retrospective analysis of patient records was carried out with caution not to link any data to patient identifiers and the confidentiality of the patient records was maintained in all instances.

Patient records were thoroughly analyzed for the following data: (1) use of intraoperative sizers or none thereof (this was confirmed by operative notes and seal of proof of sterilization stamp of a breast sizer being utilized during surgery); (2) incision location: periareolar, inframammary, or axillary; (3) plane of placement of the implants: subglandular, submuscular (including dual plane), or subfascial; (4) type and volume of implant; (5) complications such as infection, hematoma/seroma formation, and grade of capsular contracture if any.

All the patients underwent either general anesthesia or high epidural anesthesia with sedation. The surgical approach used was either inframammary or periareolar incision for augmentation mammaplasty.

Intraoperatively, all surgical pockets were irrigated by an antibiotic solution containing 1 g of cefazolin, 80 mg of gentamicin, and 500 mL of normal saline. All implants were inserted via a no-touch technique with change of gloves prior to insertion. Meticulous hemostasis was achieved in all cases but no drains were used in 410 cases of primary breast augmentation except for 12 cases where it was judged necessary intraoperatively. All cases received preoperative antibiotic of 1 g cefazolin intravenously and antibiotics (500 mg Cephalexin three times a day) were continued for 7 days postoperatively in oral form.

Incisions were closed in 3 layers for inframammary approach with 3-0 Nylon sutures placed in the subcutaneous tissues, 4-0 Nylon deep subdermal sutures, and intradermal closure with 4-0 Nylon or 4-0 Monocryl suture. For the periareolar approach, incisions were closed with 3-0 Nylon for subglandular tissue, 4-0 Nylon for subdermal sutures, and 4-0 Nylon or 4-0 Monocryl for intradermal closure. Micropore strips (3M, St. Paul, Minn.) were placed and removed 2 weeks postoperatively in both approaches.

Patients were fitted with a surgical brassiere and instructions were given to wear the apparatus for 4 weeks postoperatively. In addition, patients were told to avoid wired brassieres, lifting heavy objects over head, and strenuous physical activity for 6 weeks after surgery.

The accompanying photographs in the immediate postoperative period and at 3 and/or 6 months were also evaluated. All 416 patients were contacted by telephone or seen in person at the clinics to enquire and ascertain if any complications arose in the postoperative period. The database of patients returning for complications was also obtained from the archives at the 38th Infirmary Santa Casa Misericórdia. Patients with noted complications were questioned to enquire if any further changes from their last evaluation occurred and the primary surgeon was contacted for further information. Those who had ongoing complications were seen and evaluated at the clinic. It is postulated that over 95% of patients do return to their original surgeon for early/immediate or intermediate period postoperative complications such as hematoma, seroma, infection, and early capsular contracture. The reason is that the institute covers all revisional surgery at minimal to no cost.

## 3. Statistics

Following a normal distribution curve in both groups that underwent primary breast augmentation (sizer versus no sizer), a Student *t*-test was used to appraise overall complication rates in both groups of patients. The Central Limit Theorem tells us that the sample means are approximately normally distributed with *n* number of 416 cases (*n* = 416).

As the collated results represent multiple surgeons collectively, the paired Student *t*-test compares the difference in the means from the two variables measured on the same set of subjects to a given number (*n*), while taking into account the fact that the scores are not independent (taking into account individual surgeon variability).

The software utilized was Statistical Package for Social Studies (SPSS) (IBM Corp. Released 2012; SPSS Statistics for Windows, Version 21.0 Armonk, NY: IBM Corp.).

## 4. Results

A total of 416 patients were subjected to primary breast augmentation carried out by surgical residents from January 2011 to March 2014. These results are representative of multiple surgeon teams of the 38th Infirmary Santa Casa Misericórdia Hospital Department of Plastic and Reconstructive Surgery.

The median implant volume was 300 cc and median age of patient was 38 years (standard deviation: 8.58 years). All implants were polyurethane coated cohesive silicone gel with either round or anatomic shape. One brand was used: Silimed (Silimed, Rio de Janeiro, Brazil). Mean follow-up time was 65.30 weeks (standard deviation: 37.92) (Table 1).

The majority of implants used were round and high profile for a total of 366 cases (88.0%) and 80% (332) of these round high profile implants had a volume range within 200–300 cc. The other patients received round low profile (4 cases (1%)), round moderate profile (16 cases (3.9%)), anatomical high profile (17 cases (4%)), anatomical moderate profile (8 cases (2%)), and anatomical low profile (5 cases (0.1%)). A total of 212 cases (51%) were carried out with use of intraoperative breast sizers with the remaining 204 cases (49%) without the use of implant sizers. The breast implants were placed via periareolar approach in 210 cases (50.48%) and inframammary in 206 cases (49.52%) with the majority being placed in the subglandular pocket, 402 cases (96.6%) and 14 (3.37%) in the submuscular pocket (including dual plane.) There were no documented cases of transaxillary and transumbilical approach and subfascial placement.

Out of these 416 primary breast augmentation cases, there were 5 cases of infection (1.2%), 4 cases of seroma (1%), 3 cases of hematoma (0.7%), and 7 cases of capsular contracture (Baker's Grade III/IV) (1.7%). Total complication rate limited to infection, seroma, hematoma, and capsular contracture was 1.15% (95% CI 0.96–1.93%). There was no documented implant rupture at time of study. A limitation of this study is the time frame where cases of delayed hematoma and capsular contracture may present in the future and were not included in the study (Figure 12).

It was noted that 4 of 5 cases that were complicated by infection involved the use of intraoperative breast sizers, and that 3 out of 5 of the afflicted infections were implants placed via the periareolar approach in the subglandular plane. The remaining 2 infected cases were placed in the subglandular plane via the inframammary approach. It is interesting to note that 2 out of the 5 infections also presented with recurring seroma.

Of the cases that presented with capsular contracture (Baker's Grade III/IV), 5 out of 7 involved use of intraoperative implant sizers. All were placed subglandularly with 3 out of 5 inserted via inframammary approach and the remaining 2 via periareolar approach.

The higher rate of infection and capsular contracture in cases of primary breast augmentation whereby intraoperative sizers were used (*p* value < 0.05) is statistically significant.

### 4.1. Case Report 1

This 37-year-old patient underwent primary breast augmentation in March 2014 with 285 cc moderate profile, round cohesive polyurethane coated silicone implants inserted in the subglandular plane via an inframammary approach. Intraoperative breast sizers were utilized during surgery.

**Figure 3:**
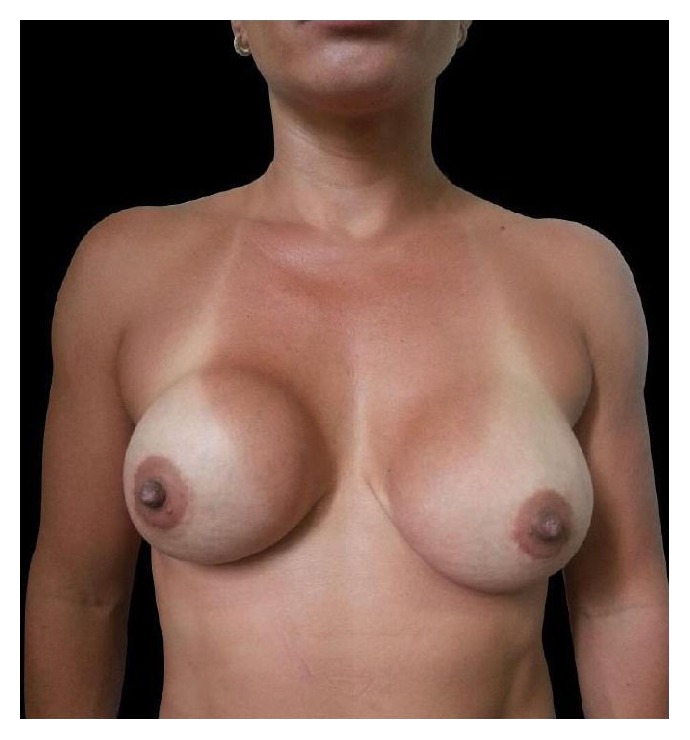
Baker Grade IV capsular contracture on right breast.

She returned in July 2014 with a Baker Grade IV capsular contracture on the right breast. The left breast was normal. The right breast was hard, painful, and grossly distorted (Figure 3).

**Figure 4:**
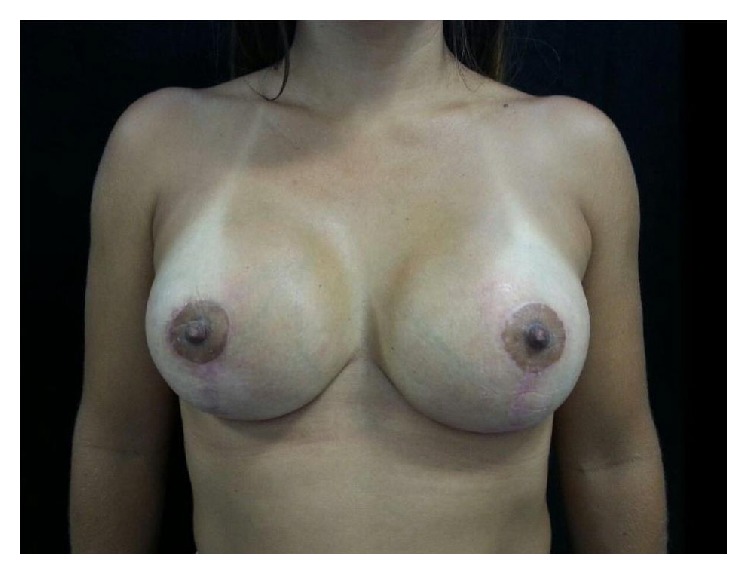
After removal of implants and augmentation mastopexy.

In December 2014, bilateral implants were removed at the request of the patient and an augmentation mastopexy was performed with 305 cc moderate profile, anatomic cohesive polyurethane coated silicone implants (Figure 4). The postoperative results were satisfactory with no early recurrence of capsular contracture to date.

### 4.2. Case Report 2

This 56-year-old lady underwent augmentation mammaplasty in September 2013 with 285 cc moderate profile, round cohesive polyurethane coated silicone implants placed in the subglandular region via the inframammary approach. Intraoperative breast sizers were used during surgery.

**Figure 5:**
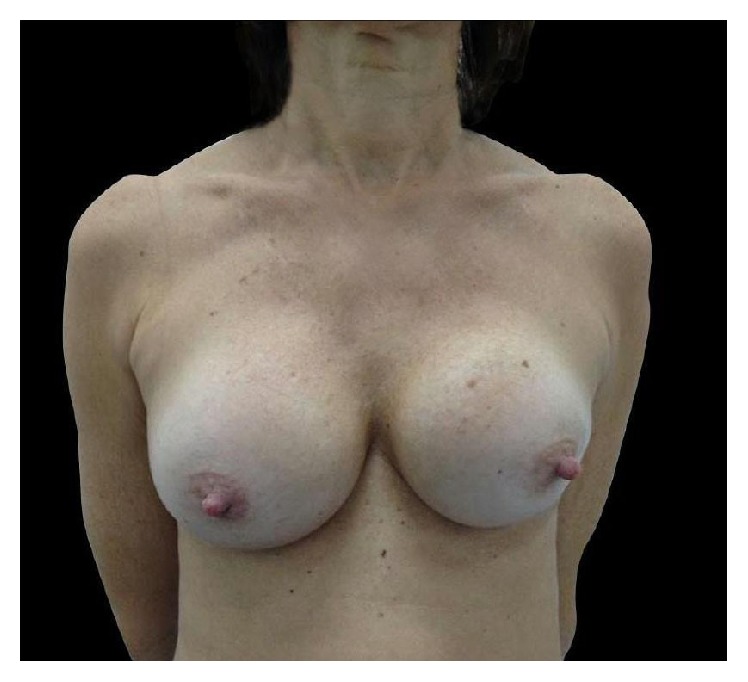
Baker Grade IV capsular contracture on left breast.

In February 2014, she noted a progressive distortion of her left breast which was painful on palpation (Figure 5). We assigned a Baker IV capsular contracture.

**Figure 6:**
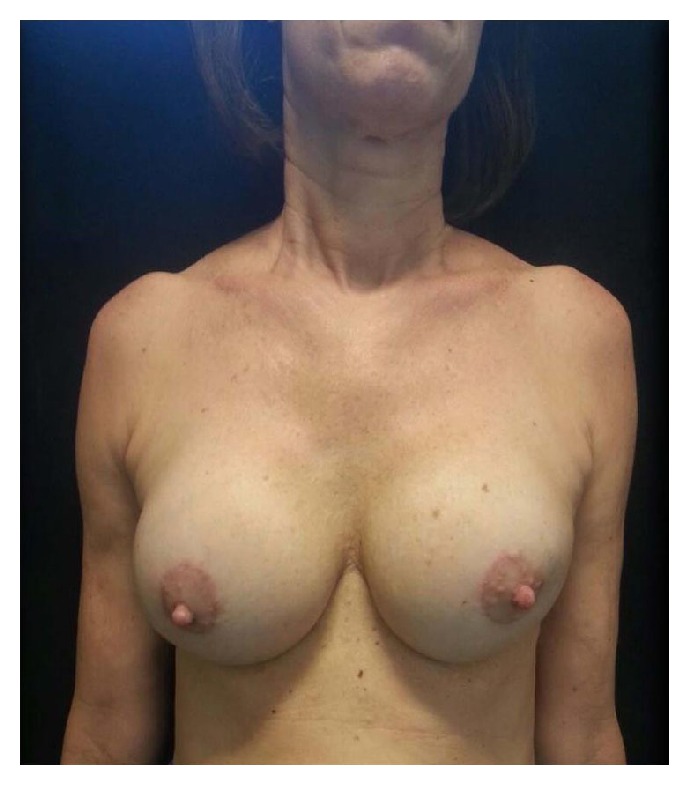
After capsulectomy and reinsertion of new 285 cc implant in subglandular plane.

The left breast implant was removed and capsulectomy was carried out with reinsertion of 285 cc polyurethane coated implants in the subglandular pocket in August 2014. Note that residual distortion is still evident postoperatively (Figure 6).

### 4.3. Case Report 3

This 35-year-old patient underwent augmentation mammaplasty in March 2013 with 275 cc high profile, anatomic cohesive polyurethane coated silicone implants inserted in the subglandular plane via a periareolar approach with use of intraoperative breast sizers.

She returned on post-op day 20 with fullness and mild serous discharge at the suture line of the periareolar regions bilaterally. Ultrasonographic assisted drainage was carried out for seroma collection. There were no systemic or local signs of infection but drained material was sent for culture and sensitivity. No implicating organisms or bacteria were isolated on cultures. Patient was started on Ciprofloxacin 500 mg twice a day and Augmentin 875 mg twice a day for 14 days.

However, the patient continued to present with recurring seroma of minimal volume (about 3 to 5 ccs on each occasion) over the next 3 months. Although we recommended removal on basis of subclinical infection, patient strongly opposed the removal. In the 3rd month, a trial of Diprospan (Betamethasone 7 mg per ampoule) was injected intramuscularly once a week for 2 weeks in an attempt to resolve inflammation. As the clinical picture improved with no more seroma, the breast implants were not removed as per request of patient. Again no particular organism was isolated on repeated culture including* Mycobacterium*.

Patient is still on 6-month follow-up and recent ultrasound scan is normal.

### 4.4. Case Report 4

**Figure 7:**
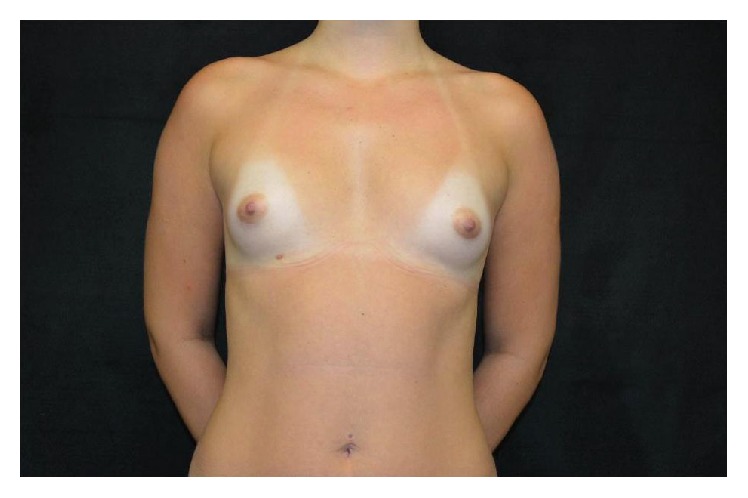
Pre-op frontal view.

**Figure 8:**
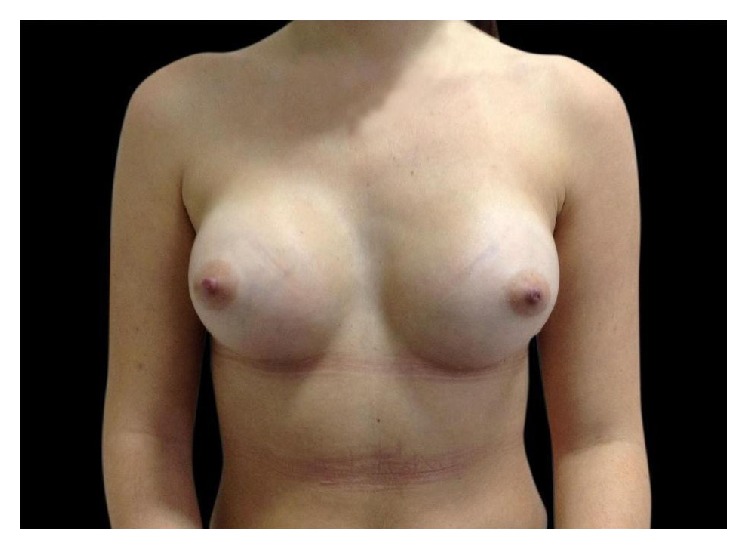
Immediate postoperative result.

This 23-year-old woman underwent primary breast augmentation in December 2013 (Figure 7). She had 280 cc high profile anatomic, cohesive polyurethane coated silicone implants inserted via the inframammary approach with intraoperative use of implant sizers. Implants were placed in the subglandular plane (Figure 8).

She returned in February 2014 (post-op day 52) complaining of redness and clear discharge from her right breast. On examination, she was hemodynamically stable and afebrile. A small fistula measuring 0.5 × 0.6 cm was noted medially with a 1 cm wound dehiscence at the lateral suture line. Antibiotic therapy with Ciprofloxacin 500 mg twice a day was initiated.

A swab was taken and 8 cc of seroma was drained via aseptic technique. The culture results returned a diagnosis of* M. abscessus*.

**Figure 9:**
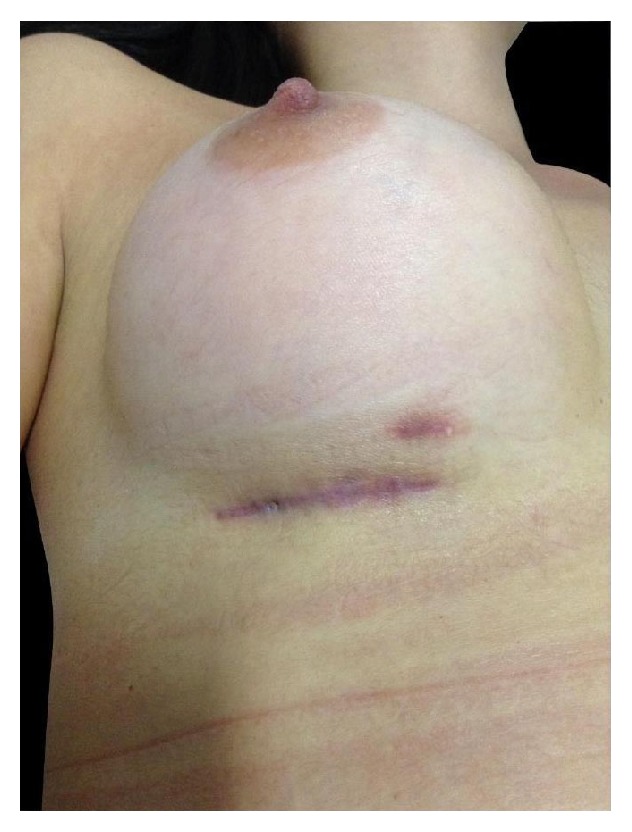
The residual fistula scar site medially.

When reexamined at the clinic on post-op day 57, it was noted that more seroma had developed and the decision was made to remove the implants surgically with debridement and commence antibiotic therapy with Clarithromycin 500 mg twice a day for 4 months (Figure 9).

**Figure 10:**
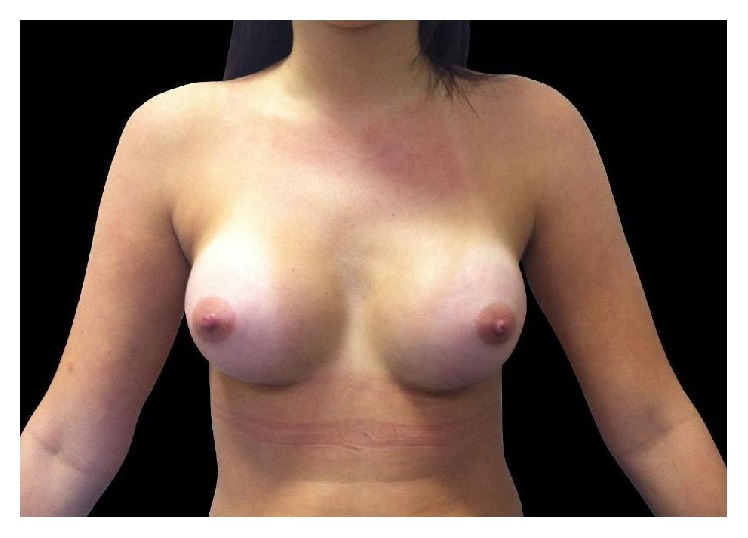
Final postoperative result.

Culture results obtained during implant removal and debridement reconfirmed the* Mycobacterium* infection. Patient subsequently had reinsertion of implant after concluding the 4 months of antibiotic therapy with satisfactory results (Figure 10).

### 4.5. Case Report 5

This 35-year-old patient underwent breast augmentation surgery in July 2012 with 265 cc bilateral moderate profile, round, cohesive, polyurethane coated silicone implants placed in the subglandular plane via the periareolar approach. Intraoperative breast sizers were utilized during surgery.

**Figure 11:**
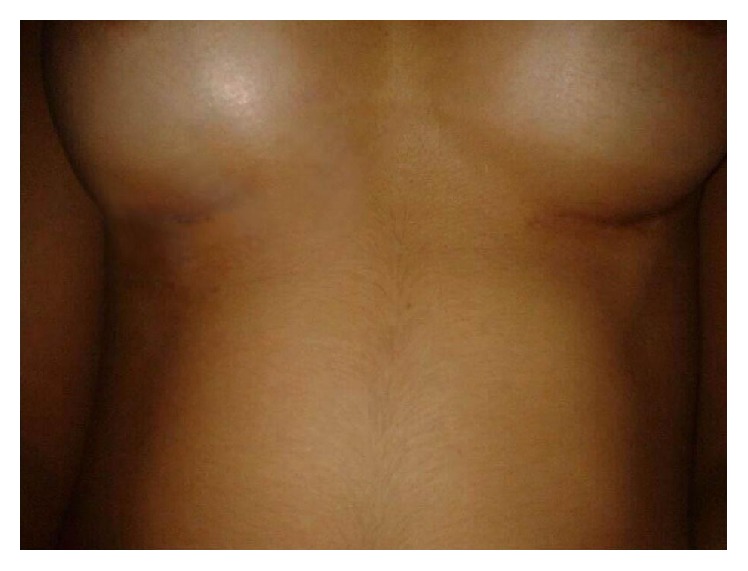
Appearance after removal of drain.

**Figure 12:**
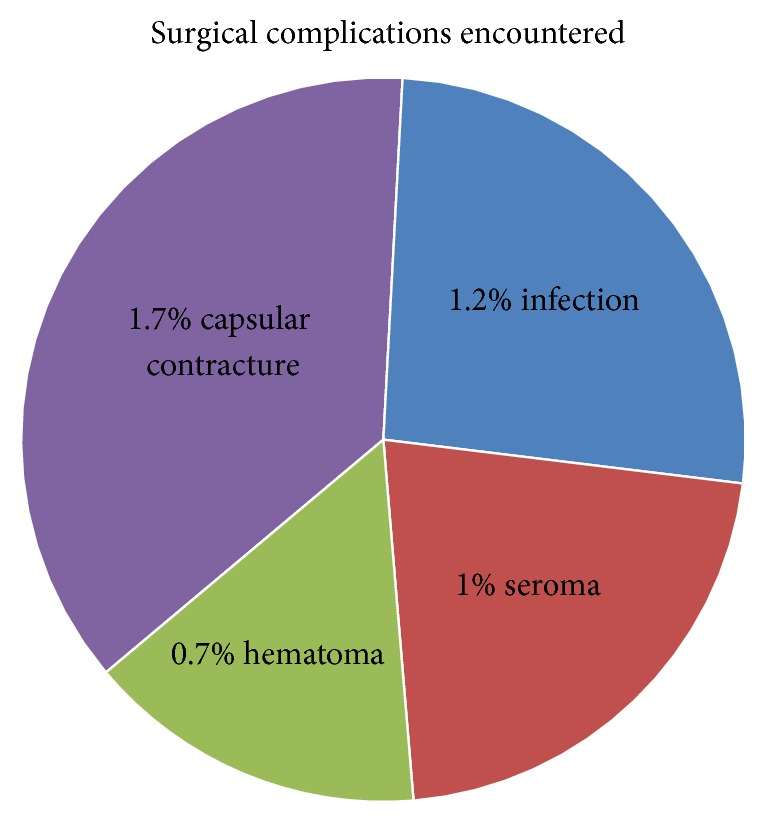
Pie chart of total percentage of surgical complications encountered.

The postoperative results were satisfactory but patient presented with a hematoma on post-op day 5. The right breast was grossly swollen and tense especially on the lower lateral region. This was drained aseptically and compression dressing was placed. The drain was removed 2 days later (Figure 11).

In August 2013, she returned with redness overlying the right breast at the previous suture line. No discharge was noted. She was prescribed Ciprofloxacin 500 mg twice a day for 7 days and the redness resolved. She has since been free of any complications and is happy with the postsurgical results.

## 5. Discussion

The exclusive use of polyurethane breast implants is both a strength and a limitation of this study. While this reduces the probability of the implant type being a confounding variable for postoperative results unrelated to the procedure, it may also account for the relative lower rates of capsular contracture among the patient group as a whole. Although selection bias was eliminated by including all patients who underwent primary breast augmentation without any other ancillary procedures within the time frame in a single institution, there would be technical variability as the cases recorded are not performed by a single surgeon.

**Figure 13:**
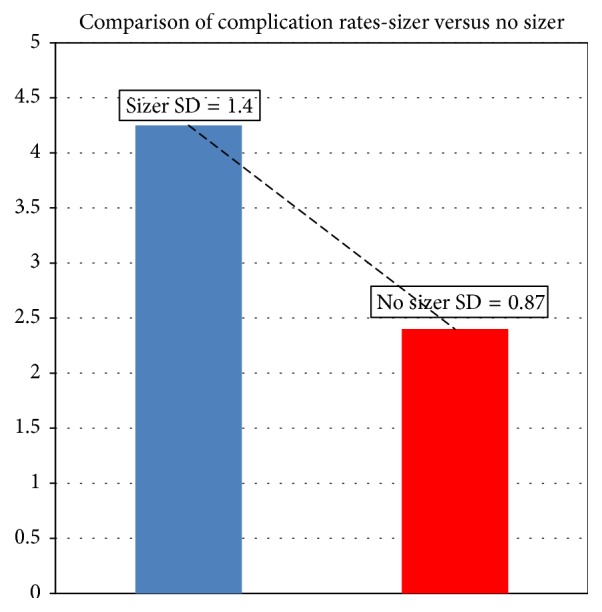
Comparison of complication rates: sizer versus no sizer. Note: *t*(8) = 2.79; *p* = 0.018.

Paired Student *t*-test was utilized to compare complication rate in surgeries with breast sizers and in those where no sizers were used. There was a significant difference in the scores for breast sizers (M = 4.3, SD = 1.4) and no breast sizers (M = 2.3, SD = 0.87) conditions, *t*(8) = 2.79, *p* = 0.018. These results suggest that the use of implant sizers is correlated with a higher complication rate in terms of infection, seroma, and capsular contracture (Figure 13).

Hence, while the results point toward a higher rate of infection, seroma, and capsular contracture in patients who underwent augmentation mammaplasty with intraoperative implant sizers (*p* < 0.05), they do not address the variability in incision site selection, size of implant, and technical variability of the surgeon.

It is critical to address the controversy of not using intraoperative breast sizers instead of using them. Many proponents of using breast sizers intraoperatively cited that the sizers allowed them to pick an appropriate size for a patient while being able to simulate the end result. Intraoperative breast sizers are also a valuable tool in a training program to allow the novice surgeon to visualize and dissect an accurate breast pocket. The critics state that the use of these intraoperative breast implant sizers unnecessarily increases tissue trauma and augments possible infection rate while not actually reflecting the final result accurately as the projection, base diameter, and cohesivity of the sizer may not mimic the chosen implant.

Some surgeons advocate a biodimensional method of selecting appropriate breast implants for a particular individual [4–6]. This method involves an objective assessment to match an implant specifically to what the breast tissue can accommodate. In such instances, the decision for a specific sized implant, base diameter, height, and projection can be determined preoperatively without the need to resort to intraoperative sizers [4–6]. In published and peer-reviewed series, there are 2500 primary breast augmentations performed with similar concepts in tissue based preoperative planning, with reoperation rates of 3% with 6 to 7 years' follow-up, compared to the reoperation rate of 15% to 20% in 3 years [7–10].

Implant sizers may augment contamination and infection risks, costs, tissue trauma, and operative time and are perhaps unnecessary in primary breast augmentation, if a surgeon is willing to quantitate tissue characteristics and use proved processes and biodimensional systems such as the High Five*™* System [6] during preoperative planning and implant selection.

Implants sizers may not accurately simulate the shape surface characteristics of the implant and in turn do not depict the appearance of a similar volume implant [4]. Sizer use can be habit forming, and surgeons who insist on using sizers rarely learn to use quantitative systems that are much more accurate and less traumatic to the soft tissues as the definite implant is only inserted once.

Notwithstanding, intraoperative breast implant sizers have their advantages as they can allow the novice surgeon to obtain an idea of an appropriate breast volume for picking an implant. They also free the surgeon from being rigid and having to learn and utilize a tissue based preoperative planning system. Proponents also claim that breast sizers are sterile, reusable, and readily available. However, health and safety regulations in the United Kingdom currently mandate that the sizers are for single use only, further increasing the cost of their use for estimating ideal implant size.

The controversy also arises if sizers do increase the rate of infection. An argument is made that biofilm accumulates on repeated use of implant sizers and this could be a problem for repeated usage [13]. Many surgeons in Asia, Latin America, and the United States resterilize their implant sizers for reuse. This may contribute to a higher probability of infection or subclinical infection.

Biofilm is a microbial community characterized by cells that are attached to a surface or to each other and that are embedded in a matrix they have produced. The biofilm possesses a highly effective defense barrier. Bacterial cells in the biofilm are protected from disinfectants, temperature changes, pH changes, antibiotics, and host defence in the form of the human immune system [15, 16].

Microorganisms suspended in liquid (water) are termed planktonic microorganisms. The various testing of the effects of different disinfection methods including autoclaving and use of ethylene oxide is carried out on planktonic microorganisms and not on established biofilm [17–19].

All antimicrobial activities will have the best effect on microorganisms in a planktonic phase before the biofilm will be established. Once the biofilm is well organized, it is more important to perform rigorous physical cleaning to destroy the biofilm [17, 18, 20]. Physical cleaning damages the biofilm, tears away parts of it, and removes the superficial layers of the biofilm. This will facilitate penetration of the bioburden by the disinfectants of which the most important is water, because water molecules finally will remove the bioburden from the breast sizer [17–20].

Thus, the mechanical removal of bacterial biofilms will therefore be more important than sterilization itself. Hence, while the breast sizer may be termed “sterile” on the surface after routine sterilization, once the surface material has been peeled or scraped off, biofilm may still be lying deep within the material itself. But by the same token, aggressive mechanical cleaning may damage the breast sizer and allow further deposition of biofilm between the microtears on the breast implant sizer itself that may serve as a nidus of infection if reused on another patient.

This hypothesis of biofilm on sizers may explain the outbreak caused by nontuberculous mycobacteria infection linked to breast augmentation surgery with implants in Campinas, Brazil [19]. The outbreak was caused by polyclonal strains of mycobacteria at different institutions, but no specific risk factors were found [19].

We recommend that breast sizers be mandated to single patient one-off use if used at all during breast augmentation surgery. Using breast sizers in this manner may not be economically feasible in many practices as multiple sizers may need to be placed into the breast pocket several times in order to determine the ideal volume necessitating that multiple sizers be purchased, stocked, and then discarded after a single patient surgery. Palmieri et al. reported an increase in operative time of an average of 10–15 minutes per case when using intraoperative sizing with graduated expander implant sizers [21]. This will have economic implications for the hospital and operating surgeon.

## 6. Conclusion

Although the infection rates and rates of capsular contracture remain low with the use of intraoperative breast sizers, we believe better results could be obtained without the use of breast sizers in primary breast augmentation with use of a biodimensional tissue based planning system while eliminating risks of infection and reducing intraoperative time. The advantage of not using an implant sizer may outweigh the advantage of using one provided the surgeon is willing to quantitate and measure breast tissue characteristics and utilize a proven biodimensional implant selection system in primary breast augmentation.

Notwithstanding, in a residency program breast sizers can be an excellent training tool. The sizers provided by implant manufacturers are helpful when it comes to determining the implant to be used particularly for a novice surgeon. Implants of various sizes can be simulated allowing the surgeon to dissect an accurate pocket for insertion of the implants. Another major advantage of the sizer is that it enables the surgeon to balance out differences in asymmetrical breasts with size discrepancy. When one is more experienced, one can begin to do so without using implant sizers but they still serve as a valuable adjunct for younger inexperienced surgeons.

Further studies need to be carried out to determine if subclinical infection and subsequently capsular contracture are indeed higher in patients undergoing primary breast augmentation with intraoperative sizers.

## Figures and Tables

**Table 1:** Characteristics of breast implants used.

Type of implant	Number of cases	Percentage
Round, HI-profile	366	88.0%
Round, LO-profile	4	1.0%
Round, moderate profile	16	3.9%
Anatomic, HI-profile	17	4.0%
Anatomic, moderate profile	8	2.0%
Anatomic, LO-profile	5	0.1%
Total	**416 **	**100.0% **
